# Advanced Pathways for the Preparation of Sensitive Lead(II) Ion Sensor: Ion-Imprinted versus Acid-Assisted Polymerization of 3‑Thiopheneacetic Acid with 1‑Vinylimidazole Monomers

**DOI:** 10.1021/acsomega.5c11178

**Published:** 2026-05-14

**Authors:** Vipul Vilas Kusumkar, Jan Svoboda, Ivana Šeděnková, Jiřina Hromadková, Michal Galamboš, Elena Tomšík

**Affiliations:** † 86879 Institute of Macromolecular Chemistry CAS, Heyrovského nám. 2, 162 00 Prague, Czech Republic; ‡ Faculty of Natural Sciences, Department of Nuclear Chemistry, 37864 Comenius University Bratislava, Ilkovicova 6, Mlynska dolina, 842 15 Bratislava, Slovakia

## Abstract

In this study, a sensing polymer film was obtained from the polymerization of two monomers—3-thiopheneacetic acid and 1-vinylimidazole. The polymerization process occurred through two distinct methods: *acid-assisted polymerization* and the *ion-imprinting technique*, with simultaneous deposition of the sensing film onto carbon rods. The deposition method plays a significant role in the films’ responses to Pb^2+^ ions. We detected Pb^2+^ ions in the absence and presence of interfering ions using square-wave voltammetry (SWV) and potentiometric detection. The sensing film obtained by acid-assisted polymerization (AS-P) demonstrated superior performance in comparison to the sensing film obtained by the ion-imprinting (II-P) technique. Specifically, films obtained by the AS-P process and measured using SWV techniques could detect Pb^2+^ ions in the presence of low (400 μM) and high (0.001 M) concentrations of interfering ions (Cu^2+^, Zn^2+^, and Co^2+^). Additionally, AS-P films can be used for the potentiometric detection of Pb^2+^ ions within a linear concentration range of 10 μM to 158 μM.

## Graphical abstract

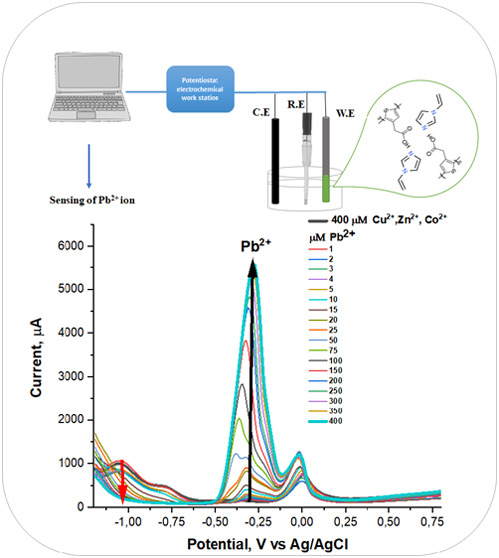

## Introduction

Lead, one of the common elements on Earth, has a special set of chemical and physical characteristics. Owing to its chemical stability, ductility, and propensity for alloy formation, the material is highly advantageous in numerous applications. These applications encompass components utilized in radiation shielding, plumbing, soldering, painting, ammunition, and battery manufacturing.^1,2^ Nevertheless, there are significant health hazards related to the ease of absorption of Pb^2+^ ions via ingestion, dermal contact, and inhalation. Upon entering the body, it rapidly disseminates and impacts numerous fundamental processes. Lead exposure is well-known to have harmful health effects and has been well studied and documented. It is known to be neurotoxic and genotoxic to humans, and it may cause cancer. In addition to causing a host of other acute and long-term health problems, it can seriously harm the central nervous system and exacerbate lung and cardiovascular dysfunctions.^3,4^ The World Health Organization (WHO) has established permissible lead exposure levels of 10 μg/l in drinking water and 0.5 μg/m^3^ in air, underscoring the necessity of stringent controls to mitigate health risks. A specialized committee on food additives has further recommended a maximum daily lead intake of 3.5 μg/kg body weight to prevent excessive accumulation in the body. Notably, experts assert that no level of lead in the bloodstream is completely without risk. Significant health consequences have been linked to even relatively low concentrations, as low as 5 μg/l.^5^ The urgent need to carefully control lead exposure to protect the public’s health is reflected in the ongoing research and regulatory initiatives.

Pb^2+^ ions can be detected using a variety of conventional analytical techniques, including atomic absorption spectroscopy (AAS),^6^ X-ray fluorescence spectrometry (XRF),^7^ inductively coupled plasma mass spectroscopy (ICP-MS),^8^ inductively coupled plasma optical emission spectrometry (ICP-OES),^9^ and atomic fluorescence spectroscopy.^10^ Unfortunately, the cost of the equipment and the complicated operations associated with these technologies often prevent their wide use. Electrochemical techniques, such as stripping voltammetry (anodic, cathodic, square wave, and differential pulse voltammetry), are recognized as valuable tools for trace analysis. These methods have several advantages, including faster analysis times, increased sensitivity and selectivity, lower costs, ease of use, and element speciation analysis capabilities.^11^

Among the electrochemical methods, stripping analysis holds particular significance as one of the most sensitive approaches for detecting trace metals. This strategy involves the use of solid electrodes to capture metal ions, followed by stripping of the electrodeposited metal. Stripping methods employing chemically modified electrodes exhibit enhanced selectivity. This is because the modifier can selectively capture specific metal ions of interest, leading to improved analytical performance.^12^

Another well-established method is potentiometry. Potentiometric detection measures the potential difference between the working and reference electrodes and offers advantages such as a wide concentration range, a low detection limit, and simplicity of use.^13^ To detect the presence of Pb^2+^ ions in targeted sources, including industrial wastewater, blood samples, and soil, various types of electrodes have been developed utilizing diverse materials.^14^ These materials encompass conducting polymers (CPs),^15^ metal oxides,^16^ carbon materials,^17^ nanoparticles,^18^ biopolymers,^19^ metal–organic frameworks,^20^ and composites.^21^ The aforementioned materials exhibit both advantages and disadvantages. The predominant challenge in the fabrication of these materials, namely, the process of preparation, is characterized by its tedious nature and substantial cost.

Conjugated polymers are useful materials for the electrochemical detection of metal ions because of their inherent ability to change the conductivity when exposed to metal ions. Polythiophenes are conjugated polymers well-known for their chemical stability and ability to form thin films on substrates. Polythiophenes, when combined with inorganic and organic compounds, can increase their sensitivity to specifically targeted metal ions.^22^

In this investigation, a cost-efficient, uncomplicated, yet efficacious method for fabricating the polymer sensing material employed in the detection of Pb^2+^ ions was devised. Acid-assisted polymerization (**AS-P**) and ion-imprinting polymerization (**II-P**) methods have been applied to synthesize a selective sensing polymer film composed of two monomers, 3-thiopheneacetic acid and 1-vinylimidazole. Both strategies have been compared for Pb^2+^ ion detection by potentiometric (**PD**) and square-wave stripping voltammetry (**SWV**) analysis methods. The physical–chemical characterization was performed using X-ray photoelectron spectroscopy, Raman spectroscopy, scanning electron microscopy, and electrochemical techniques, including cyclic voltammetry (CV) and electrochemical impedance spectroscopy (EIS).

## Experimental Part

### Materials

3-Thiophene acetic acid (3-TAA) (99.9%, Sigma-Aldrich, Czech Republic), 1-vinylimidazole (1-VIM) (99.9%, Sigma-Aldrich, Czech Republic), formic acid (Sigma-Aldrich, Czech Republic), ammonium peroxydisulfate (APS) (Lechner, Czech Republic), bithiophene (99.8%, Sigma-Aldrich, Czech Republic), and ethyl alcohol (99.8%, Sigma-Aldrich, Czech Republic) were used in the experiments as received without further purification. Ethyl alcohol (99.8%, Sigma-Aldrich, Czech Republic) and formic acid (HCOOH) were used as solvents to dissolve the chelating molecules. Lead chloride (PbCl_2_) (Sigma-Aldrich, Czech Republic), zinc chloride (ZnCl_2_), cobalt chloride (CoCl_2_), copper chloride (CuCl_2_), and sodium chloride (NaCl) (Sigma-Aldrich, Czech Republic) were used to study the sensing ability of electrodes. Carbon electrodes were used as supports and cleaned before polymer film deposition by acetone (10 min in an ultrasound bath) and ethanol (10 min in an ultrasound bath); finally, carbon electrodes were washed with distilled water and dried in air.

## Methods

The following techniques were used for the characterization of materials.

The molecular structure of the materials was studied using a Renishaw inVia Qontor Raman spectrometer equipped with 488 and 532 nm lasers and a holographic grating density of 2400 lines mm^–1^. A research-grade Leica DM 2700 M microscope with a 50× LWD Leica objective lens was used to focus the laser beam onto the sample. A Renishaw Centrus CCD detector was used to record the spectra.

X-ray photoelectron spectroscopy (XPS) measurements were performed utilizing a K-α+ spectrometer from ThermoFisher Scientific, located in East Grinstead, UK. The samples underwent analysis using a microfocused, monochromated Al Kα X-ray source possessing a spot size of 400 μm. The incident angle was set at 30° relative to the surface, while the emission angle was set perpendicular to the surface. Fitting of the XPS spectra was achieved through the application of Voigt profiles, obtained by convolving Lorentzian and Gaussian functions.

MALDI-TOF mass spectra were acquired using a Bruker MALDI-TOF spectrometer in negative-ionization mode with trans-2-3-(4-*tert*-butylphenyl)-2-methyl-2-propenylidene malononitrile as a matrix.

Scanning electron microscopy (SEM) was obtained by using a JEOL 6400 microscope.

Cyclic voltammetry (CV) and electrochemical impedance spectroscopy (EIS) were used.

The electrodes were electrochemically characterized in three-electrode cell configurations. All measurements were done at room temperature, *T* = ∼25 °C. For the counter electrode, a 1.2 cm^2^ Pt sheet was employed, and for the reference electrode, Ag/AgCl (3 M KCl) was used. Cyclic voltammetry was conducted by AUTOLAB PGSTAT302N potentiostat in an aqueous solution of H_2_SO_4_ (0.1 M) or HCl (0.1 M) between −0.1 and 0.8 V vs Ag/AgCl reference electrode with scan rates of 10 and/or 50 mV s^–1^. Electrochemical impedance spectroscopy (EIS) was performed by AUTOLAB PGSTAT302N potentiostat with a FRA32 M Module and Nova 2.2 software in an aqueous solution of HCl (0.1 M) at open circuit potential (OCP) over a frequency range of 10 kHz to 0.1 Hz with 5 mV amplitude. The Kronig–Kramers test was applied to verify the obtained EIS data.

### Deposition of Sensing Films

#### Preparation of the Dispersion of Poly-3-Thiophene Acetic Acid (Poly-3-TAA) Encapsulated with 1-VIM Using Acid-Assisted Polymerization (**AS-P**)

The acid-assisted polymerization of 3-TAA was performed at room temperature in the following manner: 0.49 g of 3-TAA (5 × 10^–1^ M), 0.049 g (1% of 3-TAA w/w) of bithiophene, 132 mg of 1-VIM (1.4 × 10^–3^ M), and 5 mL of concentrated formic acid (HCOOH). Ammonium peroxydisulfate (APS: 0.80 g in 1 mL of water, 5 × 10^–2^ M) was added, as an aqueous solution, dropwise into the mixture. The monomer-to-oxidant molar ratio was 1:0.1. After the polymerization was complete, the resulting solution was used for dip-coating the carbon electrodes (1 cm of the electrode was immersed), which were immersed in the polymerized mixture, as shown in Scheme 1a, for 2 h. The resultant electrodes were dried at room temperature. The electrodes were then dried again and used for Pb^2+^ ion sensing.

**Scheme 1:**
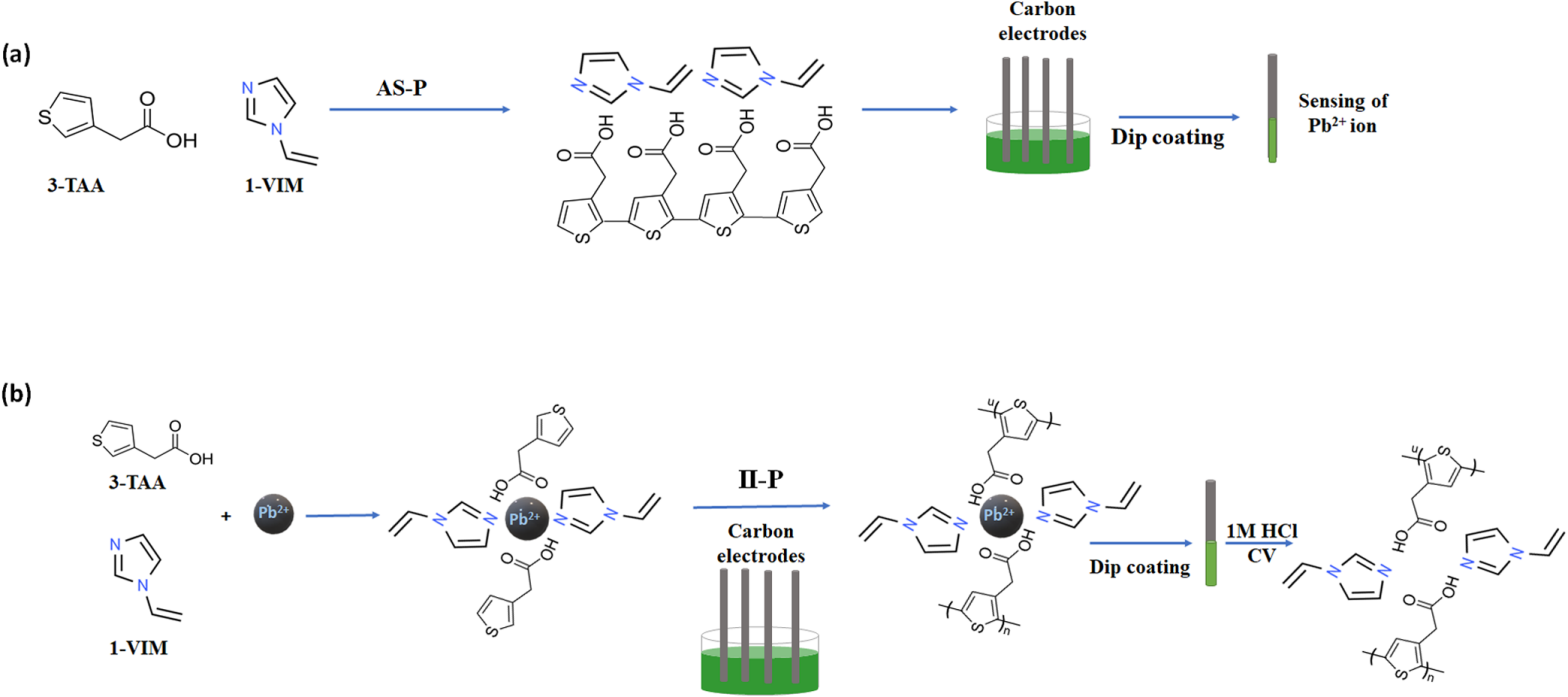
(a) Schematic Representation of the **AS-P** Method Used to Synthesize Polymer Sensing Film Based on 3-TAA/1-VIM via the Dip-Coating Method; and (b) Schematic Representation of the **II-P** Method Used to Prepare Ion-Imprinted Sensing Film Based on 3-TAA/1-VIM via the Dip Coating Method

#### Preparation of the Ion-Imprinted Polymer (**II-P**) Dispersion of Poly-3-Thiophene Acetic Acid (Poly-3-TAA) Encapsulated with 1-VIM

The acid-assisted polymerization of 3-TAA was performed at room temperature in the following manner: 0.49 g of 3-TAA (5 × 10^–1^ M), 0.049 g (1% of 3-TAA w/w) of bithiophene, 132 mg of 1-VIM (1.4 × 10^–3^ M), and 0.0735 g of Pb­(NO_3_)_2_ (2.6 × 10^–4^ M) were dissolved in 5 mL of concentrated formic acid (HCOOH). Ammonium peroxydisulfate (APS: 0.80 g in 1 mL of water, 5 × 10^–2^ M) was added, as an aqueous solution, dropwise to the mixture. The monomer-to-oxidant molar ratio was 1:0.1. After polymerization was complete, the resulting solution was used for dip-coating the carbon electrodes (1 cm), which were immersed in the polymerized mixture, as shown in Scheme 1b, for 2 h. The resultant electrodes were dried at room temperature. Subsequently, the dried electrodes were immersed in 1 M HCl solution for 24 h to remove trapped Pb^2+^ ions. The electrodes were then dried again and used for Pb^2+^ ion sensing.

### Testing

#### Potentiometry

The potentiometric measurements were conducted in a solution of 0.1 M NaCl, utilizing a 6-channel voltmeter with high input impedance (Lawson Laboratories, Malvern, PA, USA). The input impedance of this voltmeter was measured at 10^10 Ω. After the developed sensing electrodes were submerged in the solution, the studied ions were progressively added, beginning with low concentrations and gradually increasing. The solution was then stirred for 3 min, and the signal was recorded after 4 min. The measurements were carried out at room temperature, approximately 25 °C. Each measurement was repeated at least 3 times.

#### Square Wave Voltammetry

SWV measurements were conducted using a using PalmSens 4.0 Potentiostat (PalmSens Compact Electrochemical Interfaces, Netherlands) using the following steps: (a) preconditioning step: potential of −0.9 V vs Ag/AgCl for 10 s was applied before each measurement to ensure the dissolution of remaining deposits, (b) the preconcentration step proceeded at −0.9 V vs Ag/AgCl for 30 s in 0.15 M NaCl with added specific volume of lead nitrate and the solution was stirred for 3 min, and (c) the SWV was recorded from −0.8 to 0.5 V vs Ag/AgCl with 10 mV amplitude and 20 Hz frequency.

#### SWV with Interfering Ions (Low or High Concentrations)

The SWV of Pb^2+^ ions detection in the presence of interfering ions was carried out by dissolving CuCl_2_, ZnCl_2_, and CoCl_2_ with low or high concentrations: (a) low concentration – 400 × 10^–6^ M, and (b) high concentration – 0.01 M.

The SWV measurement of individual interfering ions was done, and the results are presented in the Supporting Information.

### Calculation

The linear fitting of the concentration curves was completed with Origin 2019.

## Results and Discussion

### Synthesis and Dip-Coating of the Sensing Layer

The polymer sensing layers were created by using a unique and straightforward process involving the synthesis, dip-coating, and characterization stages. This study aimed to investigate the effect of introducing the chelating ligand, 1-vinylimidazole (1-VIM), into the polymeric dispersion of poly-3-thiopheneacetic acid (poly-3-TAA) for Pb^2+^ ion detection. The study was divided into two segments. In the first study, the **AS-P** strategy was employed, where Pb^2+^ ions were not used during the preparation of the sensing film (Scheme 1a). In the second segment, the **II-P** strategy was employed, whereby Pb^2+^ ions were allowed to form a stable solution with the two ligands 3-TAA and 1-VIM, followed by polymerization conducted using APS as an initiator, as illustrated in Scheme 1B. Then, the Pb^2+^ ions were removed from the film, and the final sensing film was characterized and tested.

The roadmap of the entire workflow is shown in Figure 1. These two distinct approaches aimed to ascertain which strategy would be more advantageous for the selective detection of Pb^2+^ ions in the targeted source. Although the approaches differ, they both employ a novel method involving the preparation of the polymer sensing material achieved through the dispersion of a polymer containing poly-3-TAA encapsulated with 1-VIM. Such a strategy allows the resulting polymeric dispersion to be conveniently applied as a sensing layer. The surface of the carbon electrode (chosen as a current collector) was coated by immersing the electrode for two hours into the polymerization solution (Scheme 1a,1b) and allowing it to dry in air overnight. A minimum half of the carbon electrode surface was utilized, resulting in a straightforward yet highly effective sensing formation, as shown in Scheme 1a. More details of the film synthesis are presented in the experimental part.

**Figure 1:**
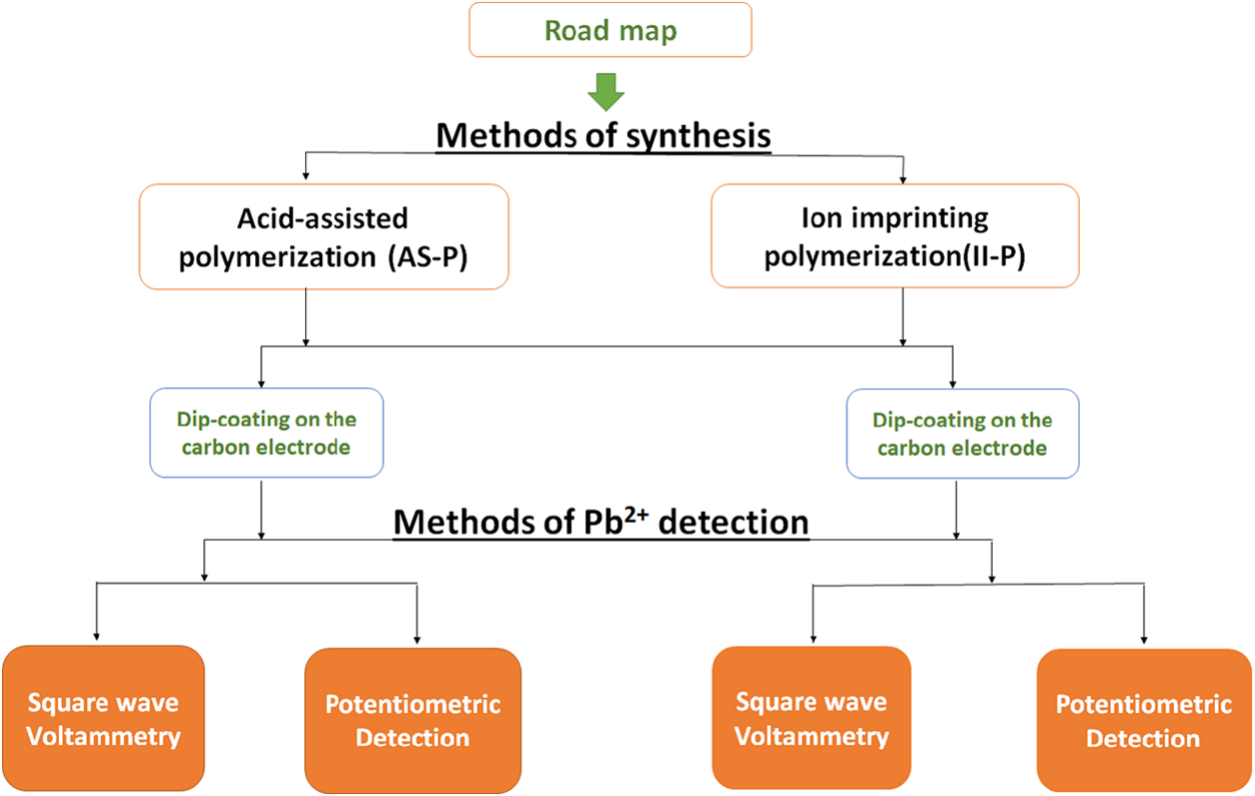
A pictorial representation of the roadmap of the synthetic methods and methods of Pb^2+^ detection.

When **II-P** was applied (see Scheme 1b), the Pb^2+^ ions were incorporated into a polymer sensing film at the beginning. The sensing films were dried in air for overnight. To remove the Pb^2+^ ions from the sensing film, CV was applied in three-electrode cell configurations in 1 M HCl. It is suggested that formed cavities (Scheme 1b), after CV treatment, could be advantageous for Pb^2+^ detection, especially when interfering ions are present.

The two detection strategies—SWV and PD are applied for at least 3 electrodes for each deposition method to check the reproducibility of the results. The choice of the detection methods is based on the fact that SWV is more accurate but demands a skillful person to perform the study. On the other hand, the PD method is simple but less accurate.

All synthesized sensing electrodes were kept in the laboratory conditions in air and were further used for investigation without special pretreatment.

### Characterization of the Material

The polymer films obtained by the **AS-P** and **II-P** methods were characterized by scanning electron microscopy, Raman and X-ray photoelectron spectroscopies, and electrochemical techniques (cyclic voltammetry and electrochemical impedance spectroscopy), and the results are presented below.

Figure 2 represents the surface morphologies of the polymer films obtained by two different methods. It is noticeable that the sensing film prepared by **AS-P** is less porous compared with the **II-P** strategy. Moreover, the image in Figure 2b represents the **II-P** film with incorporated Pb^2+^ ions from the synthesis, and Figure 2c shows that the surface of the film changed after treatment in 1 M HCl. For the detection of any ions, a porous sensing film is desirable as higher porosity facilitates ion accessibility and can lower the detection limit. SEM analysis confirms that **II-P** films possess a more porous surface morphology compared with the **AS-P** films.

**Figure 2:**
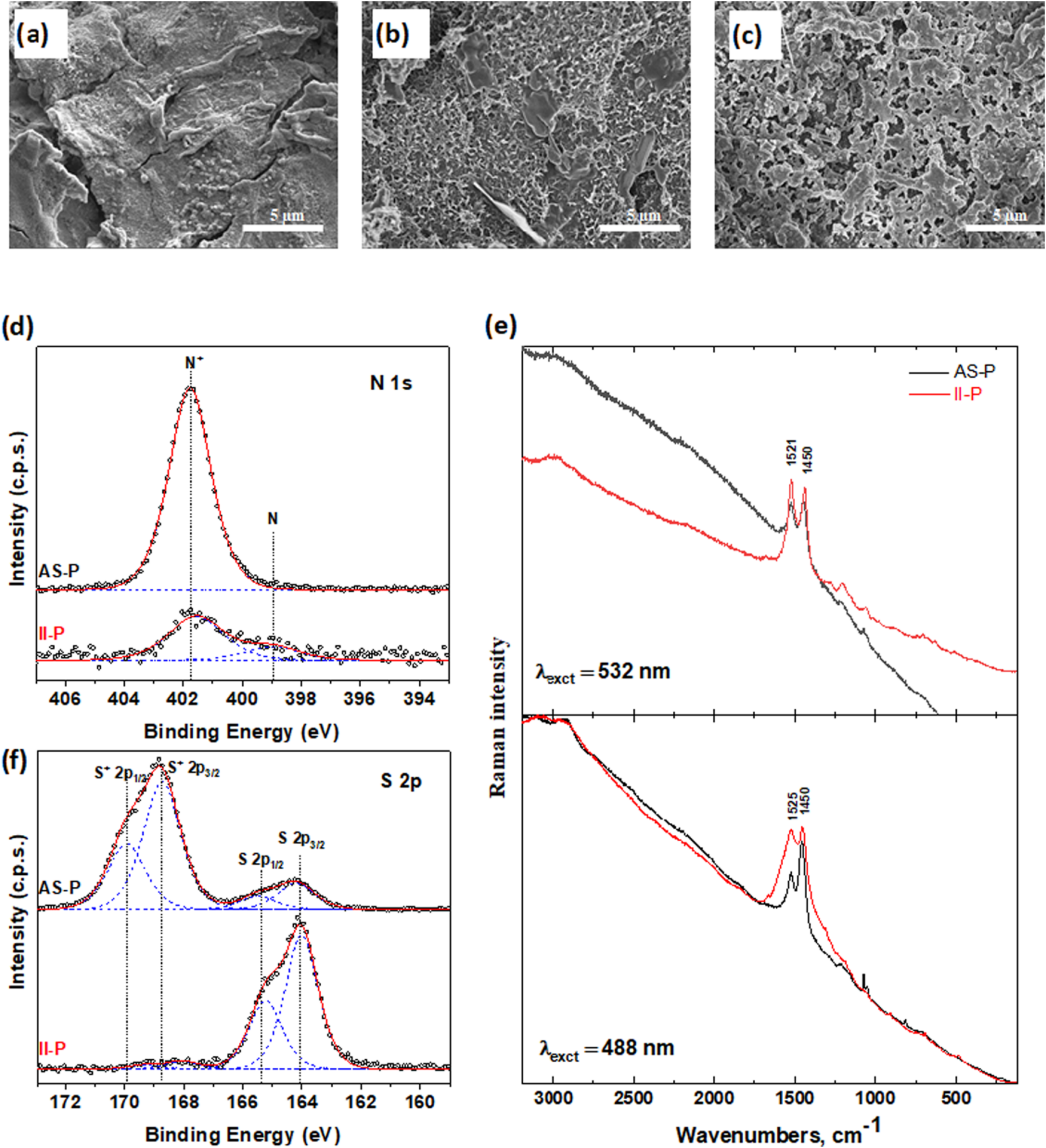
Representative SEM images of polymer sensing films obtained by (a) **AS-P** method, (b) **II-P** method with lead ions, and (c) **II-P** after removing lead ions. High-resolution XPS of (d) **AS-P** and **II-P** of N 1s; (f) S 2p; and (e) Raman spectroscopy of **AS-P** and **II-P** measured with two excitation lasers, 532 and 488 nm.

The chemical structure of the sensing films was characterized by two techniques: X-ray photoelectron spectroscopy (XPS) and Raman spectroscopy, and the corresponding results are presented in Figure 2. The survey XPS spectra are provided in Figure S1.

The high-resolution XPS data for the N 1s (confirming the presence of 1-VIM monomer units) and S 2p (confirming the presence of 3-TAA monomer units) for **AS-P** and **II-P** films are summarized in Figure 2d,f, respectively. The data presented for the **II-P** film were collected after the removal of Pb^2+^ ions; the corresponding spectra of the **II-P** film prior to washing are shown in Figure S2 in the SI. Notably, the surface concentration of the N 1s atoms is higher for the film prepared by the **AS-P** method than for those obtained via the **II-P** approach, indicating a greater surface abundance of 1-VIM monomer units in the films obtained by the **AS-P** method. Furthermore, the polymer film prepared by the **AS-P** method exhibits sulfur atoms from the 3-TAA monomer in both the neutral and charged states (Figure 2d). In contrast, only neutral sulfur atoms were detected in the **II-P** films.

The Raman spectra of **AS-P** and **II-P** films, measured using two different excitation lasers, are similar and are presented in Figure 2e. Two dominant bands observed in the spectra of both **II-P** and **AS-P** samples can be attributed to vibrations of the 1-VIM and 3-TAA rings. The band at 1525 cm^–1^ is assigned to the C–N stretching vibration in the imidazole ring^23^ and to the C = C stretching vibration in the thiophene ring.^24^ Although the intensity of the bands is slightly dependent on the wavelength of the excitation, the comparison of the spectra measured with the green and blue lasers confirms the higher concentration of 1-VIM in the sample **II-P**. However, this funding does not corroborate the XPS results (see Figure 2d), suggesting that both techniques are required for accurate confirmation of the chemical structure of the composite film. In addition, the Raman spectra of **AS-P** films reveal the presence of unreacted APS when excited at 488 nm (see Figure S5 in the SI). The presence of characteristic graphite spectral features in the Raman spectra, obtained with both excitation wavelengths, further confirms the thin nature of the **AS-P** films on the electrode surface compared to the thicker films obtained by the **II-P** method (Figures S4 and S5 in the SI).

### Electrochemical Characterization

Electrochemical characterization was performed using CV and EIS to find the difference in electrochemical activity of the sensing films obtained by two methods. Figure 3a shows the CV responses recorded in 0.2 M HCl over a potential range of 0–0.8 V vs Ag/AgCl 3 M KCl reference electrode at a scan rate of 10 mV/s. The measured currents were normalized to the geometric surface area of the electrodes.

**Figure 3:**
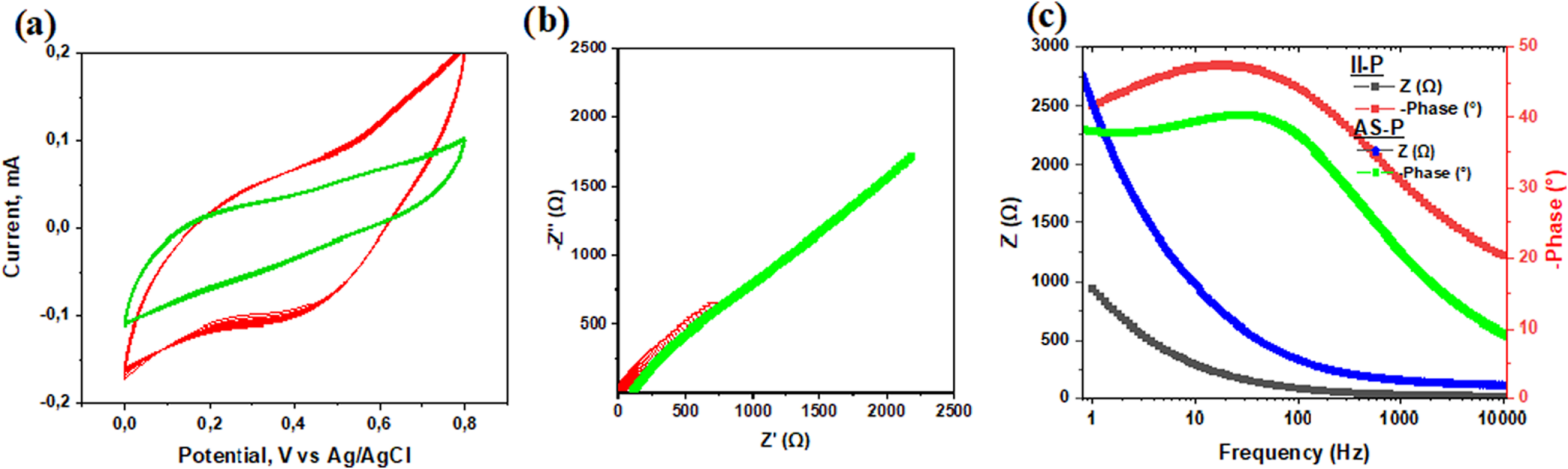
Graphs depicting (a) CV for **AS-P** electrodes (green) and **II-P** films (red), (b) Nyquist plots for **AS-P** electrodes (green) and **II-P** films (red), and (c) Bode plots for **AS-P** electrodes (blue and green) and **II-P** films (black and red).

The CV data clearly indicate that the sensing film obtained by the **II-P** method exhibits higher current responses than the film prepared by the **AS-P** method. Furthermore, the EIS results demonstrate that the impedance (real and imaginary parts) is smaller for the **II-P** film compared to **AS-P** film, presented in b. The approximately 5-fold higher impedance observed for the **AS-P** film could be attributed to its film morphology (see a). The compact and less porous structure of the **AS-P** films hinders ion penetration into the films, which is consistent with the CV results.

Notably, the Bode plots (Figure 3c) reveal similar phase-angle responses as a function of applied frequency for sensing films obtained by both methods (red and green curves). In both cases, the phase-angle reaches a maximum at approximately 60 Hz, corresponding to a characteristic time constant of ∼0,02 s.

### Testing

#### Detection of Pb^2+^ Ions Without the Presence of the Interfering Ions

To evaluate and confirm the detection capability of the prepared sensing polymer films, two independent techniques—potentiometry detection (PD) and square-wave voltammetry (SWV)—were applied, and the results obtained from both methods were compared.

Figure 4a,4b present the potentiometric detection of Pb^2+^ ions using sensing films prepared *via* the **AS-P** or **II-P** methods. The **AS-P** film exhibits a cationic response, with the measured electromotive force (EMV) increasing as the Pb^2+^ concentration rises. A key performance metric for potentiometric sensors is the detection limit, which was determined by fitting the linear Nernstian region of the curve in Figure 4a (red line).

**Figure 4:**
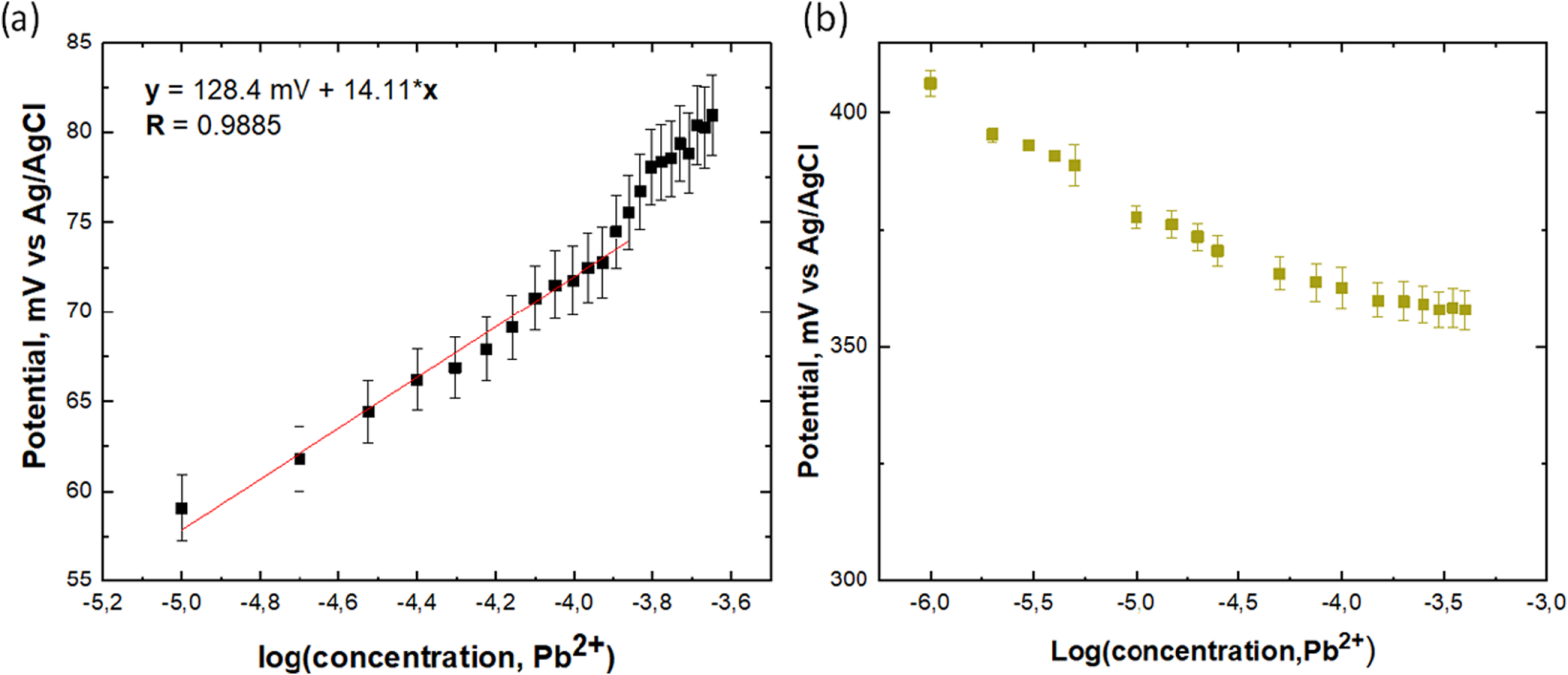
Response curves of the potentiometric detection of Pb^2+^ ions by (a) **AS-P** and (b) **II-P** sensing films.

From the fitting data, the relationship between EMV and log­[Pb^2+^] was determined, yielding a slope of 14.11 mV per decade of concentration, which is less compared to the theoretical Nernstian slope of 29.5 mV expected for bivalent cations. The **AS-P** sensing film exhibits approximately half this value for Pb^2+^ ions, which can be attributed to the chemical composition of the film and its surface morphology. The detectable concentration range for the **AS-P** sensing film extends from 10 μM to 158 μM. In contrast, the sensing film obtained by the **II-P** method did not detect Pb^2+^ ions at all (see Figure 4b), only a slight decrease of EMV vs added Pb^2+^ ions were observed. This behavior may be related to the porous structure of the **II-P** film, which allows Cl^–^ ions to penetrate and partially screen the Pb^2+^ response.

Based on the results obtained using the PD method—namely, the narrow detection range of the sensing film prepared by the **AS-P** method and the lack of the potentiometric response for the **II-P** sensing films—it was decided to apply the SWV method to evaluate the developed sensing films. Figure 6 presents the data for the individual determination of Pb^2+^ ions using by the SWV method over the concentration range of 1 μM to 400 μM in 0.15 M NaCl solution, measured in a three-electrode cell configuration. The sensing electrodes obtained by the **AS-P** method (Figure 6a) exhibit a linear response in the range from 5 μM to 300 μM, as indicated by the fitting curve shown in Figure 6b.

The metal ions were reduced at constant potential before determination (detailed procedure could be found in the experimental part). The anodic peak current was measured at −0.57 V vs Ag/AgCl reference electrode and was observed to shift slightly with increasing Pb^2+^ concentration. Moreover, a slight shift in the peak position toward a more positive potential, from −0.57 to −0.45 V vs Ag/AgCl, was observed with increasing Pb^2+^ concentration. This behavior can be attributed to the IR drop across the polymer film.^25,26^

The individual determination of Pb^2+^ ions using the sensing film prepared via the **II-P** method was carried out by SWV, and the results are presented in Figure 5c. The anodic peak current was measured at −0.48 V vs Ag/AgCl and also shifted toward more positive potential with increasing Pb^2+^ concentration, reaching −0.35 V vs Ag/AgCl. The corresponding calibration curve is shown in Figure 5d and reveals a linear response in the concentration from 2 μM to 80 μM range (see insert in Figure 5c for details). Notably, the total current recorded for the electrodes obtained by the **II-P** method is higher compared to the current for the **AS-P** electrodes. This behavior can be explained by the more porous structure of the polymer films formed via the **II-P** method. Moreover, CWV curves recorded for **II-P** electrodes are broader than those obtained for the **AS-P** electrodes. Based on these results, it can be concluded that sensing films obtained by the **AS-P** method exhibit superior performance compared to those electrodes prepared by the **II-P** method.

**Figure 5:**
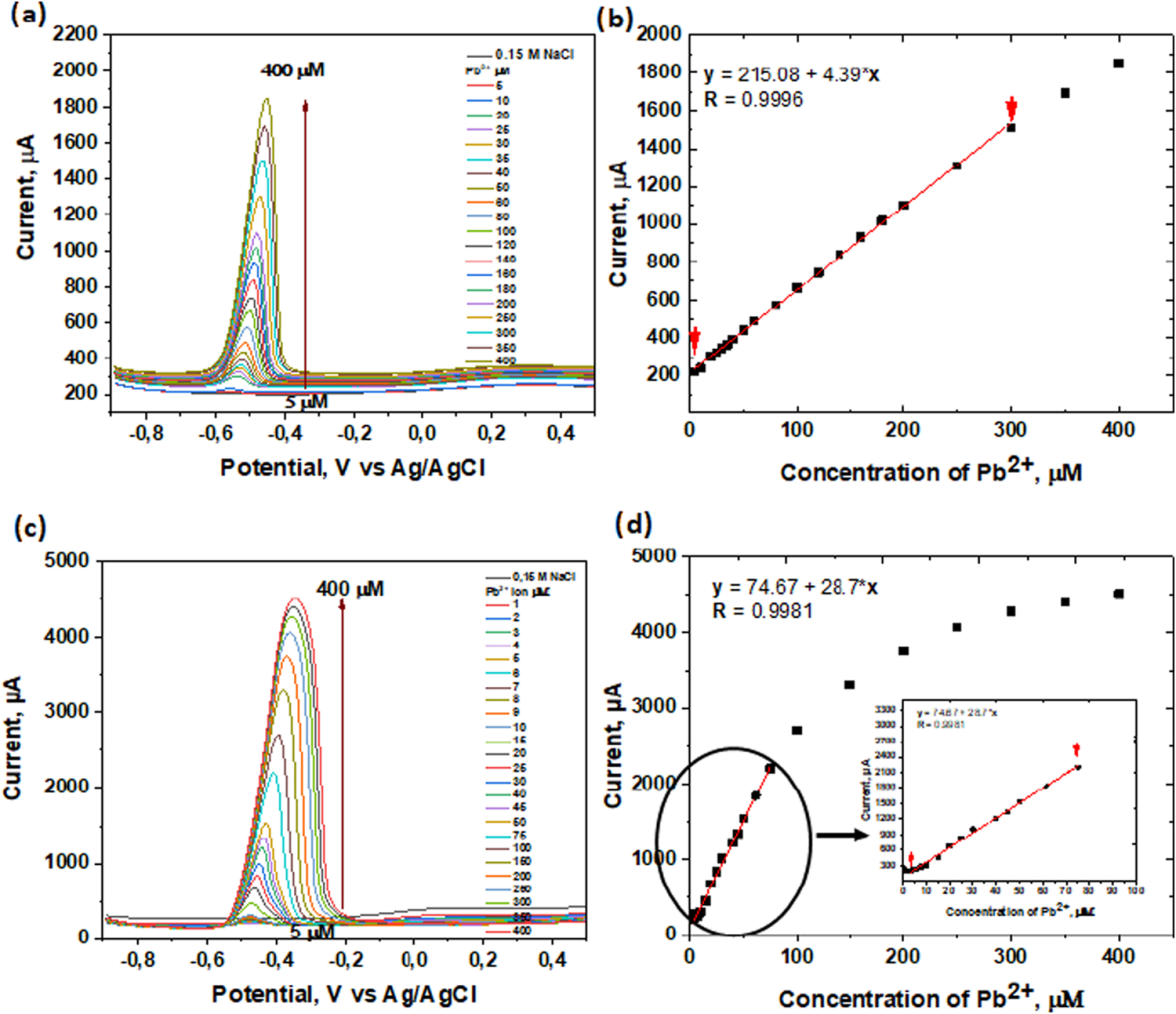
SWV measurements for the different concentrations of Pb^2+^ ions in 0.15 M NaCl solution for the **AS-P** electrodes (a, b) and **II-P** electrode (c, d).

#### Detection of Pb^2+^ Ions in the Presence of Interfering Ions (Low and High Concentrations)

To further assess the selectivity of the sensing films prepared by **AS-P** and **II-P** methods, their response toward Pb^2+^ ions was investigated in the presence of potentially interfering metal ions (Cu^2+^, Zn^2+^, and Co^2+^), as shown in Figure 6. Measurements were carried out at two interfering-ion concentrations: low (400 μM) and high (0.001 M). The lower concentration (400 μM) was selected to match the concentration of Pb^2+^, thereby simulating competitive conditions at equal ion levels. The higher concentration (0.001 M) was chosen to create a more demanding environment in which the interfering ions were present in excess relative to Pb^2+^. This approach enables a comprehensive evaluation of the electrode selectivity under both moderate and extreme interference conditions.

**Figure 6:**
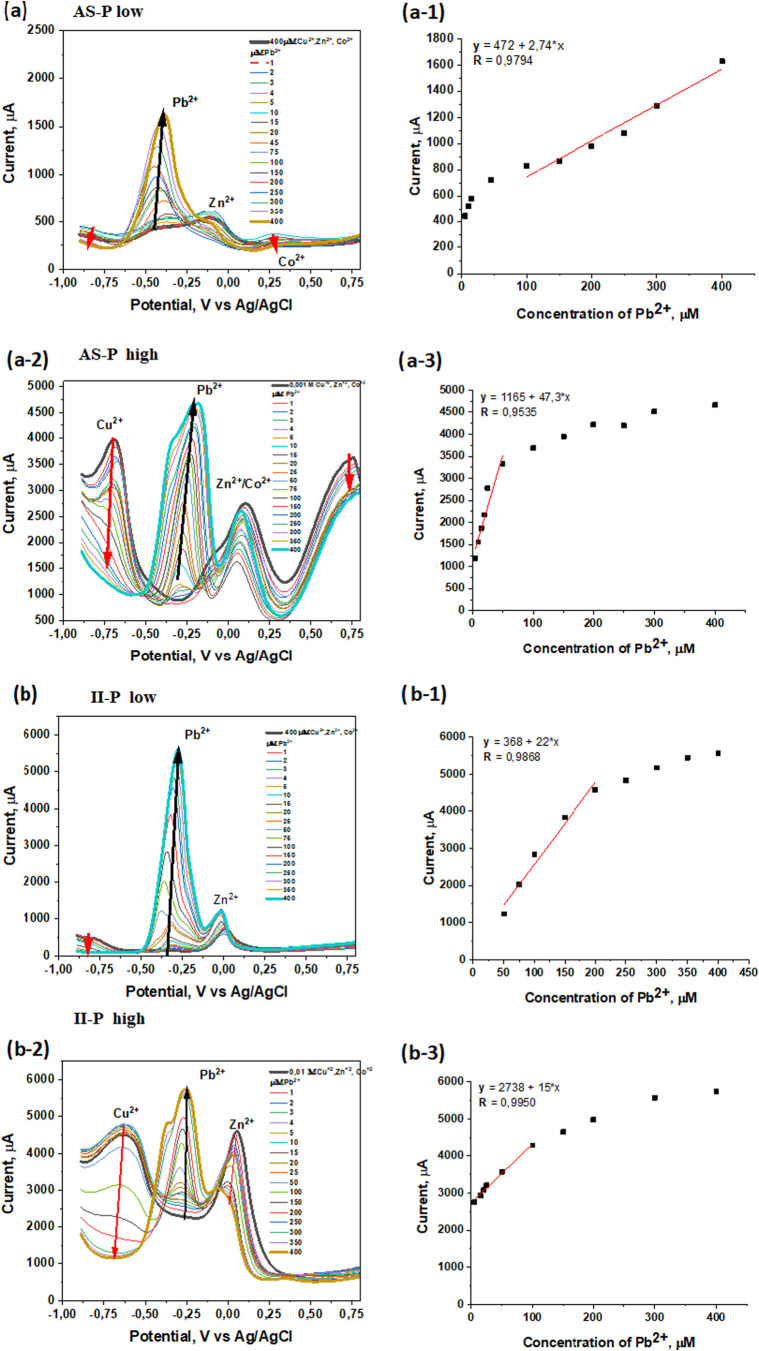
Detection of Pb^2+^ ions by the SWV method in the presence of low or high concentrations of CuCl_2_, CoCl_2_, and ZnCl_2_ as interfering ions for sensing film obtained by (a) **AS-P** and (b) **II-P** methods.

In addition, the responses of the prepared sensing film toward individual Cu^2+^, Zn^2+^, and Co^2+^ ions were also recorded separately and are presented in Figure S5 in the SI for comparison. According to the literature, these ions are among the primary interfering species that may affect the electrochemical detection of Pb^2+^. Therefore, their inclusion in the selectivity study provides a realistic assessment of the practical applicability of the developed sensing films.

A solution containing 400 μM of Cu^2+^, Zn^2+^, and Co^2+^ ions in 0.15 M NaCl was prepared and used for the detection of Pb^2+^ ions with **AS-P** electrodes (see Figure 6a). The results demonstrate the increase in the anodic peak current with increasing Pb^2+^ concentration. The presence of interfering ions at this low concentration does not significantly deteriorate the response of the **AS-P** electrodes toward Pb^2+^ ions. For a Pb^2+^ concentration of 5 μM, the anodic peak current is recorded at −0.4 V vs Ag/AgCl reference electrode, and its position remains nearly unchanged with increasing Pb^2+^ ion concentration. However, lower Pb^2+^ concentration (from 1 to 4 μM) could not be reliably detected due to signal overlap.

For comparison, the anodic peak potential of the individual interfering ions is located at −0.45 V vs Ag/AgCl for Cu^2+^ ions (Figure S6a, SI), 0.0 V vs Ag/AgCl for Zn^2+^ ions (Figure S5c, SI), and −0.45 V vs Ag/AgCl for Co^2+^ ions (Figure S5e, SI).

In the mixture solution at high interfering-ion concentration (0.001 M), the potential peak for the interfering ions shifted to −0.55 V vs Ag/AgCl for the Cu^2+^, −0.13 V vs Ag/AgCl for Zn^2+^ ions, and 0.25 V vs Ag/AgCl for Co^2+^ ions (Figure 6a). The corresponding calibration plot (Figure 6a-1) exhibits a linear response in the 100 to 400 μM range.

The anodic peak for Pb^2+^ in this high-interference environment was recorded at −0.30 V vs Ag/AgCl and is clearly distinguishable from the interfering-ion peaks (Figure 6a-2). As the Pb^2+^ concentration increases, the peak currents of the interfering ions decrease, while the Pb^2+^ peak increases, resulting in a linear calibration plot for Pb^2+^ in the 5–75 μM range (Figure 6a-3).

Overall, these results demonstrate that the **AS-P** electrodes are capable of reliably detecting Pb^2+^ ions at both low and high concentrations of potentially interfering ions, confirming their high selectivity under varying conditions.

Similarly, the **II-P** electrodes were evaluated, and the results are shown in Figure 6b. The anodic current recorded for the **II–P** electrodes is higher than that of the **AS-P** electrodes, which can be attributed to the more porous morphology of the **II-P** sensing film. The corresponding calibration plot at low interfering-ion concentration is presented in Figure 6b-1, indicating that the **II-P** electrodes can detect Pb^2+^ in the range of 50–200 μM; at lower Pb^2+^ concentrations, the anodic peaks overlap with those of the interfering ions. In the presence of high interfering-ion concentrations, the detection behavior of the **II-P** electrodes is similar to that of the **AS-P** electrodes, with a linear response for Pb^2+^ in the range of 5–100 μM. Notably, the anodic peak potentials for the **II-P** electrodes are shifted toward more positive values compared to the **AS-P** electrodes, which is related to both the morphology of the polymer films and the ratio of 1-VIM to 3-TAA in the sensing layers. Based on the **SWV** measurements, it can be concluded that both **AS-P** and **II-P** electrodes are suitable for the selective detection of Pb^2+^ ions.

To compare our findings with the published data, we summarized them in the presented Table 1.

**Table 1:** Summary of the Published Results of Modified Electrodes for the Determination of Pb^2+^ ^a^

type of sensing layer	detection method	linear range (M)	ref
PPy-CO_2_@PGEj	DPASV	0.1 × 10^–9^ to 1.0 × 10^–9^	^25^
graphene/bismuth nanocomposites	DPASV	1 × 10^–6^ to 100 × 10^–6^ g/L	^26^
Fe_3_O_4_/MWCNTs/LSG/CS/GCE	SWASV	1 × 10^–6^ to 0.2 × 10^–3^	^27^
N/IL/G/SPCE	SWASV	0.1 × 10^–9^ to 0.1 × 10^–6^	^28^
GO@Fe_3_O_4_@CBT/GCE	SWASV	0.3 × 10^–9^ to 72 × 10^–9^	^29^
MWCNTs-PARS/GCE	DPASV	5 × 10^–6^ to 150 × 10^–6^	^30^
L-CSPTE	SWASV	1 × 10^–6^ to 200 × 10^–6^	^31^
Hg-Bi/PDAAQ/GCE	DPV	10 × 10^–9^ to 0.12 × 10^–6^	^32^
poly(AN-*co*-HAS)	potentiometry	1 × 10^–10^ to 1 × 10^–3^	^33^
**AS-P** electrodes	SWV	5 × 10^–6^ to 0.3 × 10^–3^	this work

a DPASV: differential pulse anodic stripping voltammetry; DPV: differential pulse voltammetry; SWASV: square wave anodic stripping voltammetry; SWV: square wave voltammetry.

The detection method is crucial for the accurate determination of the target ions (Table 1). Furthermore, the choice of the sensing film plays an important role in Pb^2+^ detection, as previously reported.^25–33^ The linear range of the different types of sensing films, the long-term stability of the sensors, and their selectivity straightforwardly depend on the chemical composition of these films. An ultrasensitive Pb^2+^ potentiometric sensor based on copolyaniline nanoparticles has been reported, exhibiting a long lifetime.^33^

In the current work, the **AS-P** electrodes were prepared using a novel acid-assisted polymerization method (which is considered a green method)^34,35^ that can be applied for Pb^2+^ detection by two detection strategies: **SWV** and **PD**. The **AS-P** electrodes exhibit superior detection toward Pb^2+^ ions by the **SWV** method, even in the presence of low and high concentrations of interfering ions.

## Conclusion

We have devised a cost-efficient, uncomplicated, yet efficacious method for fabricating the polymer sensing material employed in the detection of Pb^2+^ ions. We have applied acid-assisted polymerization (**AS-P**) and ion imprinting polymerization (**II-P**) methods to synthesize a selective sensing polymer film composed of two monomers, 3-thiopheneacetic acid and 1-vinylimidazole, and compared both strategies for Pb^2+^ ions detection by potentiometric (**PD**) and square-wave stripping voltammetry (**SWV**) analysis methods. The **AS-P** electrodes exhibit a wider linear range at low interfering-ion concentrations (5–300 μM) and provide clearly distinguishable peaks even in the presence of both low and high concentrations of Cu^2+^, Zn^2+^, and Co^2+^ ions. The **II-P** electrodes show higher overall anodic currents due to their more porous morphology, but their low-concentration detection range is slightly narrower (50–200 μM). Under high interference conditions, both electrode types maintain reliable detection of Pb^2+^, with linear ranges of 5–75 μM (**AS-P**) and 5–100 μM (**II-P**). Peak potential shifts observed in **II-P** electrodes toward more positive values reflect differences in polymer composition and film structure. Overall, the data indicate that both electrode types are suitable for Pb^2+^ sensing, with **AS-P** electrodes being preferable for low-concentration detection and **II-P** electrodes offering enhanced current responses.

## Supplementary Material



## Notes

Original data of XPS measurements; XPS spectra of the polymer film obtained by **II-P** before the removal of Pb^2+^ ions; Response curves of the potentiometric detection of Pb2+ ions; Raman spectra by laser 532 nm; Raman spectra by laser 488 nm; the SWV measurement of AS-P and **II-P** films of individual metal ions; dependence of measured current from SWV measurements vs concentration (PDF)
