# Supplementary material for: Advanced Pathways for the Preparation of Sensitive Lead(II) Ion Sensor: Ion-Imprinted versus Acid-Assisted Polymerization of 3‑Thiopheneacetic Acid with 1‑Vinylimidazole Monomers
Source: ACS Omega. 2026 May 14;11(20):29488–97. doi: 10.1021/acsomega.5c11178 (PMC13216980; doi:10.1021/acsomega.5c11178)
Supplement: Supplementary file 1 [file ao5c11178_si_001.pdf]

Supporting Information

Advanced Pathways For Preparation of Sensitive Lead (II) Ion Sensor: Ion-Imprinted versus Acid-Assisted Polymerization of 3-Thiopheneacetic Acid with 1-Vinylimidazole Monomers

*Vipul Vilas Kusumkar[a,b], Jan Svoboda[a], Ivana Šeděnková[a], Jiřina Hromadková[a], Michal Galamboš [b], Elena Tomšík[a] **

[a]*Institute of Macromolecular Chemistry CAS, Heyrovského nám. 2, 162 00 Prague, Czech Republic*

[b]*Comenius University Bratislava, Faculty of Natural Sciences, Department of Nuclear Chemistry, Ilkovicova 6, Mlynska dolina, 842 15 Bratislava, Slovakia*

Email: tomsik@imc.cas.cz.

Content

Original data of XPS measurements S-3

XPS spectra of the polymer film obtained by **II-P** before the removal of Pb^{2+} ions S-4

Response curves of the potentiometric detection of Pb^{2+} ions S-5

Raman spectra by laser 532 nm S-6

Raman spectra by laser 488 nm S-7

The **SWV** measurement of **AS-P** and **II-P** films of individual metal ions S-8

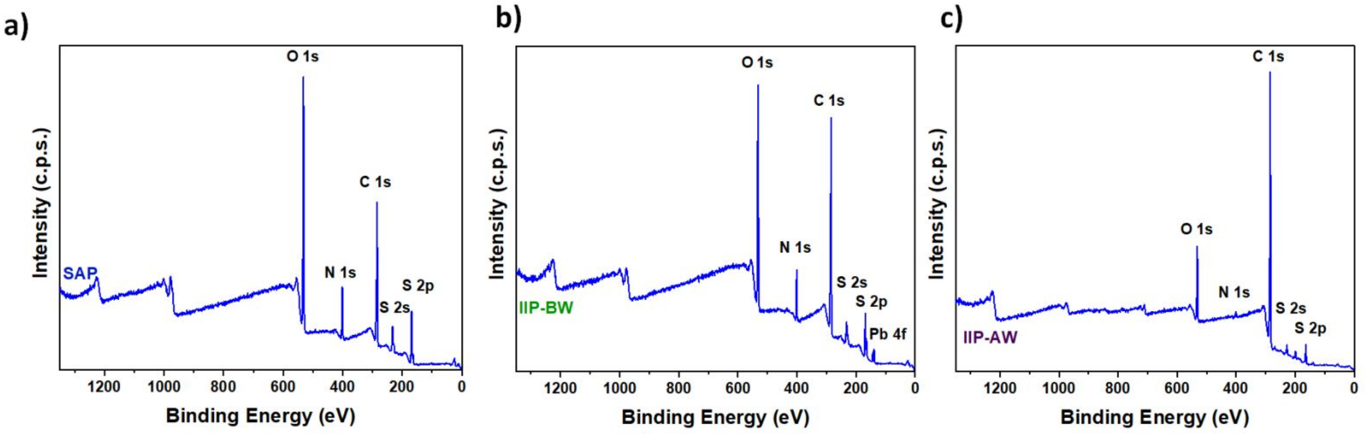

Figure S1. Original data of XPS measurements for a) polymer film obtained by **AS-P**; b) polymer film obtained by **II-P** with Pb^{2+} (II-P-BW), and c) polymer film obtained by **II-P** without Pb^{2+} (II-P-AW).

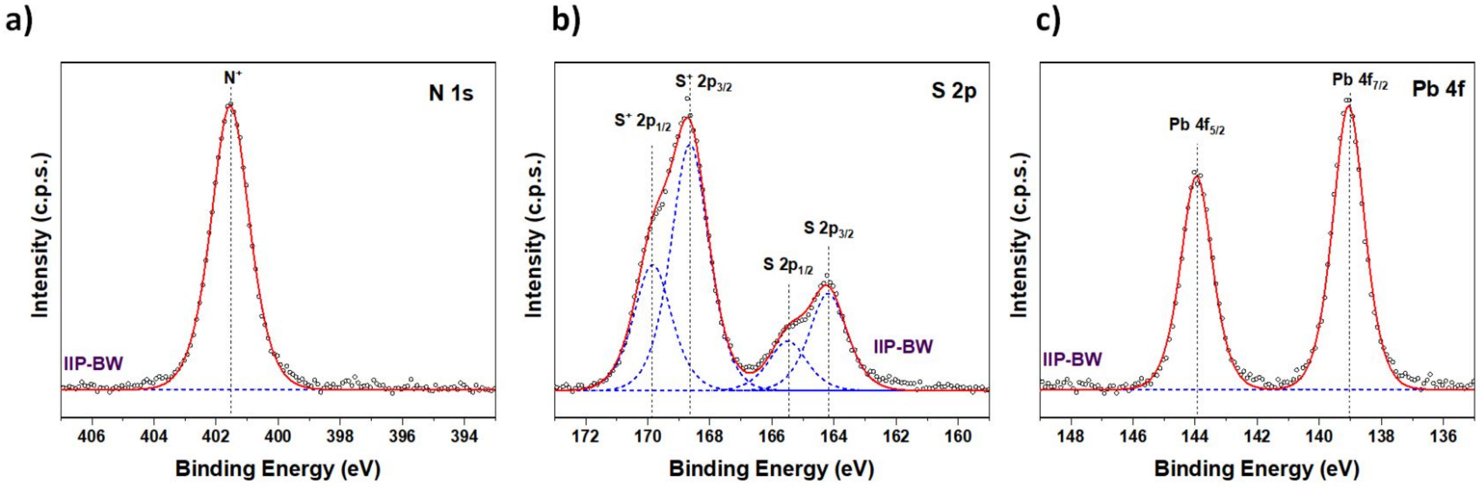

Figure S2. XPS spectra of the polymer film obtained by **II-P** before the removal of Pb^{2+} ions: a) N 1s, b) S 2p, and c) Pb 4f.

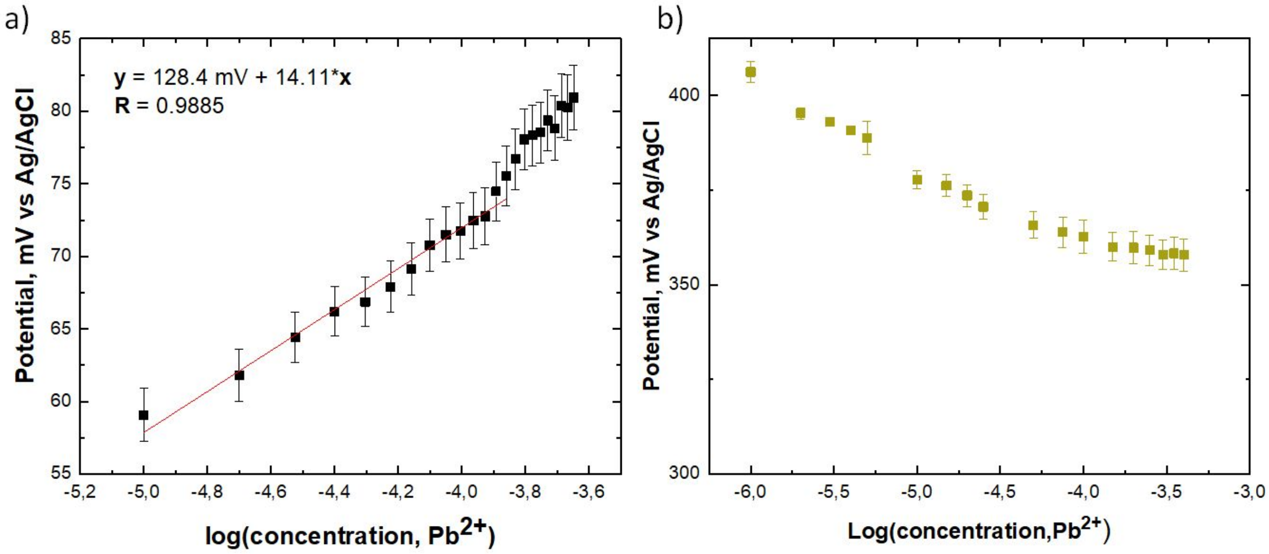

Figure S3. Response curves of the potentiometric detection of Pb^{2+} ions by a) **AS-P** and b) **II-P** sensing films.

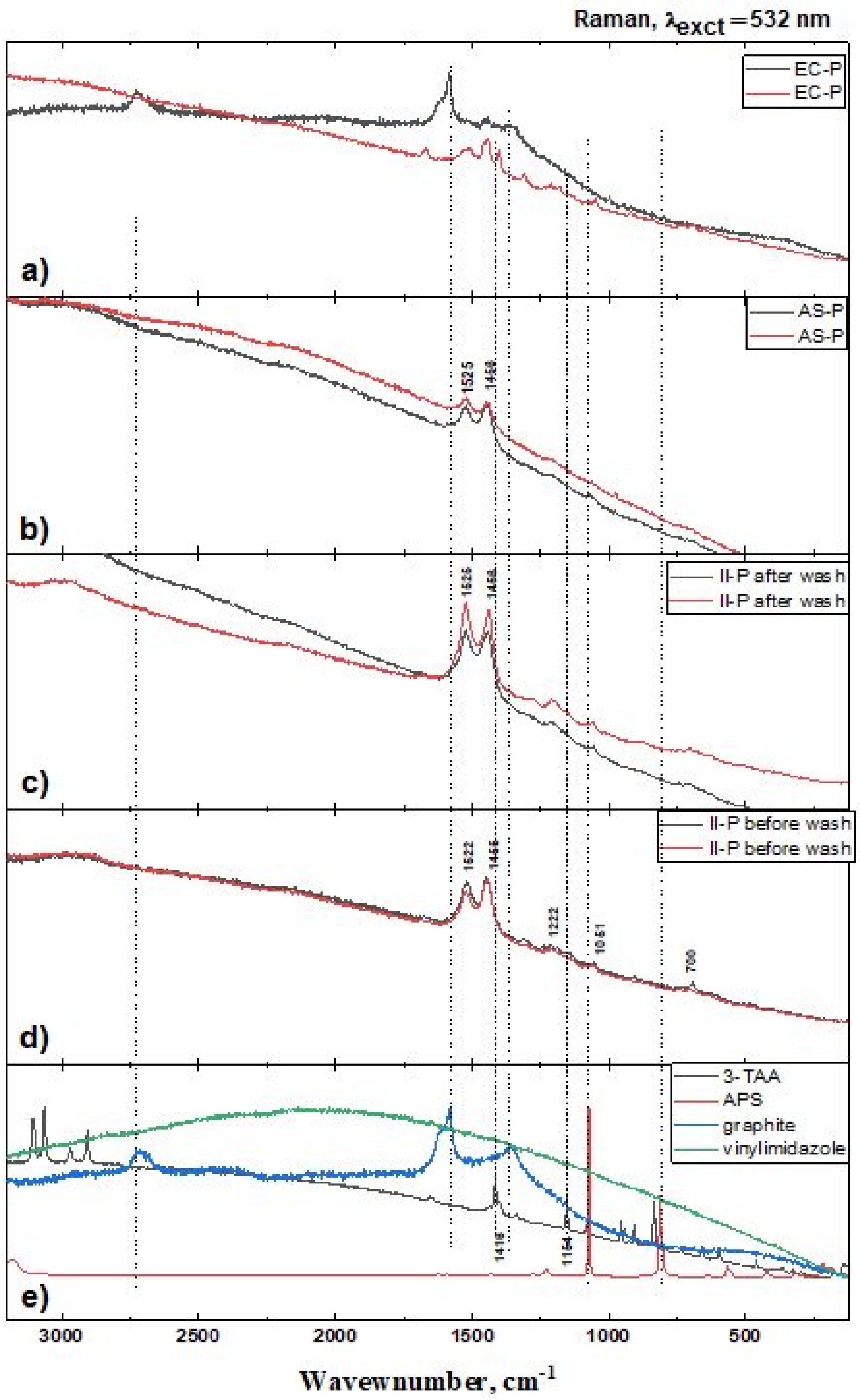

Figure S4. Raman spectra by laser 532 nm for: a) electrochemically deposited polymer, b) AS-P, c) II-P without Pb^{2+} ions, d) II-P with Pb^{2+} ions, and e) monomers and support.

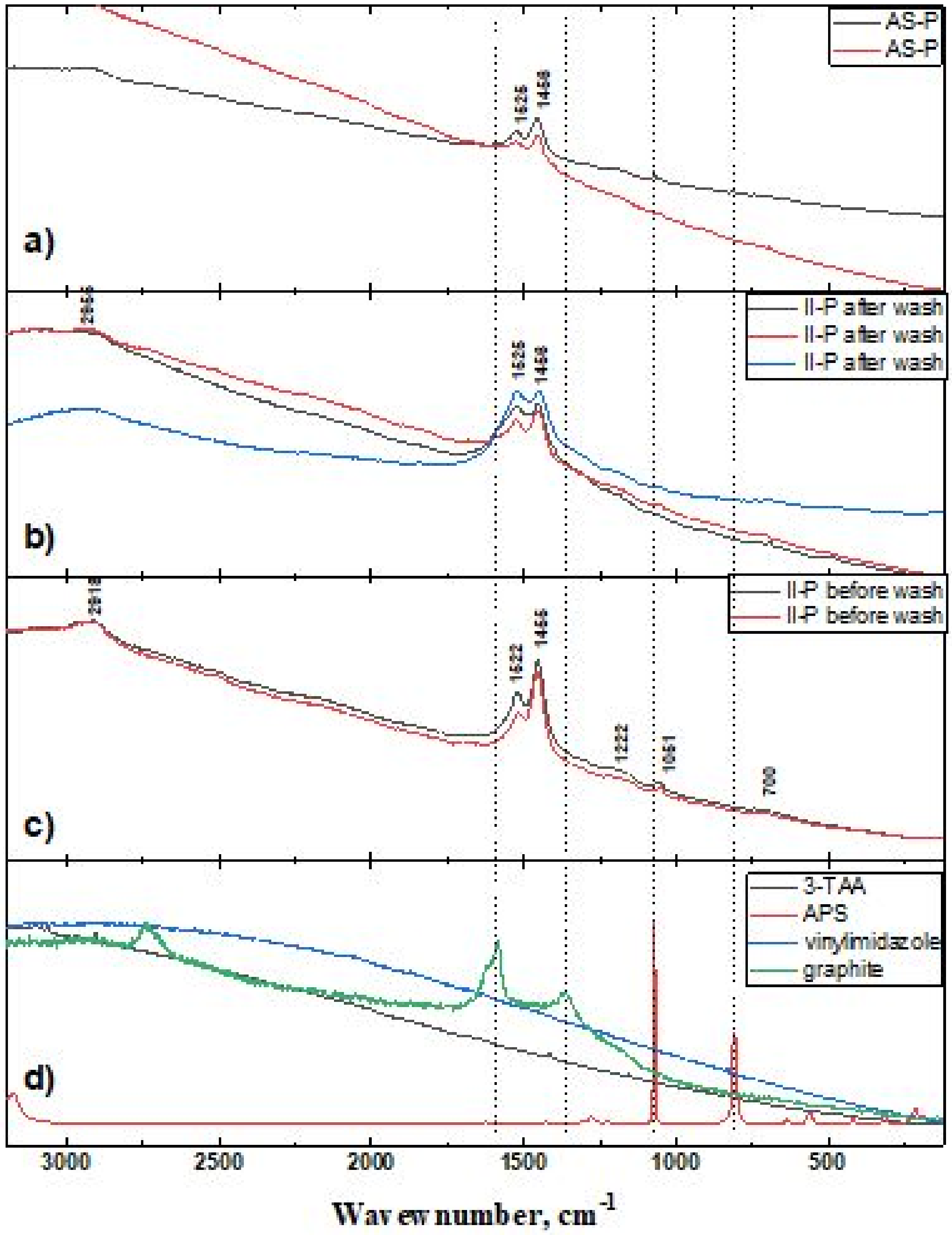

Figure S5. Raman spectra by laser 488 nm for: a) AS-P, c) II-P without Pb^{2+} ions, and d) II-P with Pb2+ ions, and e) monomers and support.

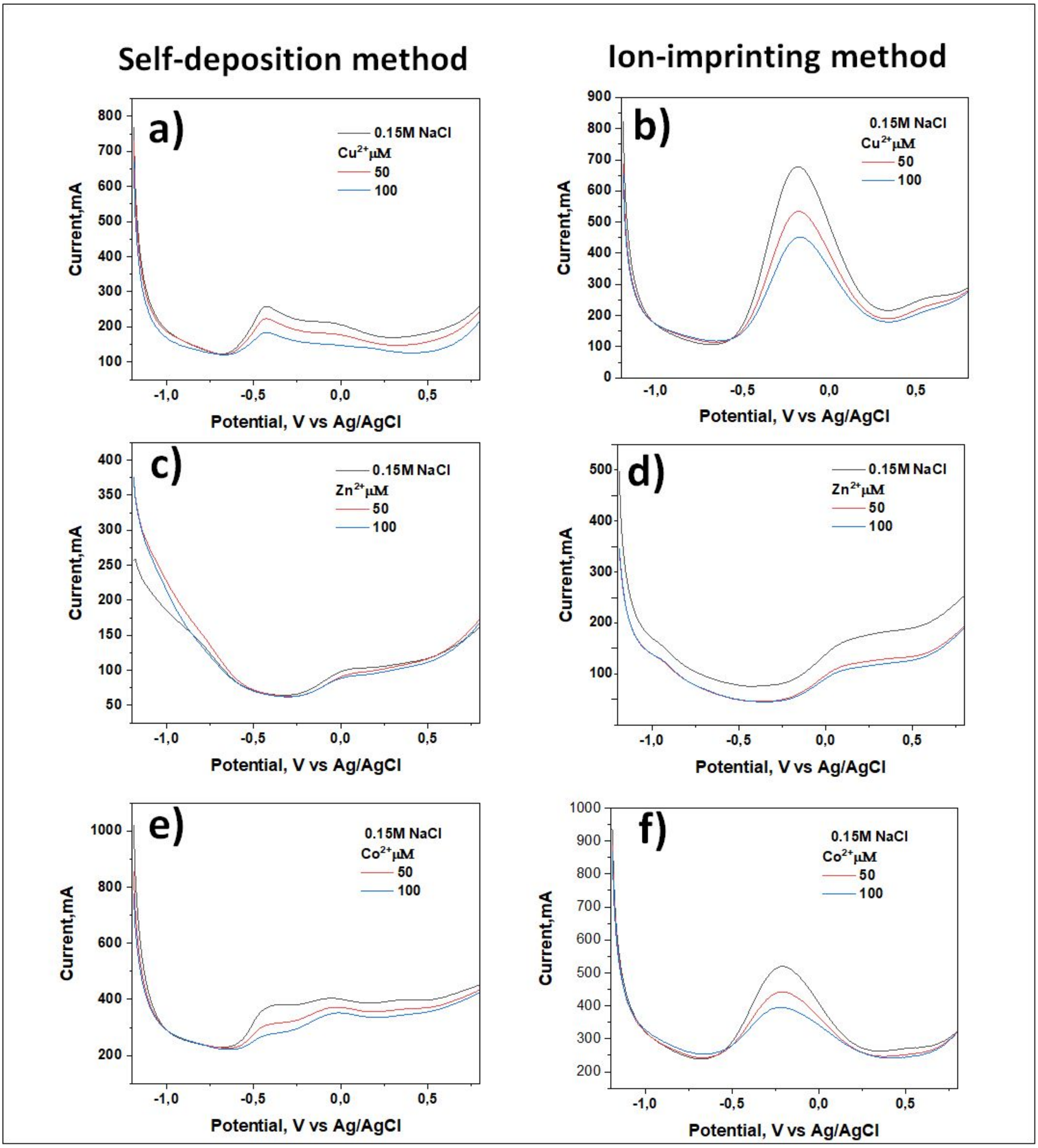

Figure S6. The SWV measurement of **AS-P** and **II-P** films of individual metal ions: a) and b) Cu^{2+} ions; c) and d) Zn^{2+} ions; e) and f) Co^{2+} ions.
